# Identification of key genes and mechanisms of epicardial adipose tissue in patients with diabetes through bioinformatic analysis

**DOI:** 10.3389/fcvm.2022.927397

**Published:** 2022-09-09

**Authors:** Huiping Yang, Bingquan Xiong, Tianhua Xiong, Dinghui Wang, Wenlong Yu, Bin Liu, Qiang She

**Affiliations:** Department of Cardiology, The Second Affiliated Hospital of Chongqing Medical University, Chongqing, China

**Keywords:** bioinformatic analysis, diabetic, EAT, IL-1β, CD274, inflammatory response, Nf-κB, immune infiltration

## Abstract

**Background:**

In recent years, peri-organ fat has emerged as a diagnostic and therapeutic target in metabolic diseases, including diabetes mellitus. Here, we performed a comprehensive analysis of epicardial adipose tissue (EAT) transcriptome expression differences between diabetic and non-diabetic participants and explored the possible mechanisms using various bioinformatic tools.

**Methods:**

RNA-seq datasets GSE108971 and GSE179455 for EAT between diabetic and non-diabetic patients were obtained from the public functional genomics database Gene Expression Omnibus (GEO). The differentially expressed genes (DEGs) were identified using the R package DESeq2, then Gene Ontology (GO) and Kyoto Encyclopedia of Genes and Genomes (KEGG) pathway enrichment were analyzed. Next, a PPI (protein–protein interaction) network was constructed, and hub genes were mined using STRING and Cytoscape. Additionally, CIBERSORT was used to analyze the immune cell infiltration, and key transcription factors were predicted based on ChEA3.

**Results:**

By comparing EAT samples between diabetic and non-diabetic patients, a total of 238 DEGs were identified, including 161 upregulated genes and 77 downregulated genes. A total of 10 genes (IL-1β, CD274, PDCD1, ITGAX, PRDM1, LAG3, TNFRSF18, CCL20, IL1RN, and SPP1) were selected as hub genes. GO and KEGG analysis showed that DEGs were mainly enriched in the inflammatory response and cytokine activity. Immune cell infiltration analysis indicated that macrophage M2 and T cells CD4 memory resting accounted for the largest proportion of these immune cells. CSRNP1, RELB, NFKB2, SNAI1, and FOSB were detected as potential transcription factors.

**Conclusion:**

Comprehensive bioinformatic analysis was used to compare the difference in EAT between diabetic and non-diabetic patients. Several hub genes, transcription factors, and immune cell infiltration were identified. Diabetic EAT is significantly different in the inflammatory response and cytokine activity. These findings may provide new targets for the diagnosis and treatment of diabetes, as well as reduce potential cardiovascular complications in diabetic patients through EAT modification.

## Introduction

Diabetes mellitus is a chronic metabolic disease characterized by hyperglycemia and defective or resistant insulin secretion. As people’s dietary habits and lifestyles change, the prevalence of diabetes continues to rise (1). A previous epidemiological study revealed that approximately 462 million people had type 2 diabetes (T2DM) in 2017, which is equivalent to 6.28% of the world’s population (2), and more than 1 million deaths can be attributed to diabetes every year, making it the ninth leading cause of death. Meanwhile, people with diabetes have a higher risk of cardiovascular disease (3). Obesity, hypercholesterolemia, hypertriglyceridemia, and elevated blood pressure are considered major risk factors for combined cardiovascular disease in patients with diabetes (4).

Epicardial adipose tissue (EAT), a brown fat tissue (5) located between the epicardium and myocardium and directly surrounding the coronary arteries (6), is an active endocrine organ (7). EAT can synthesize and secrete adipokines, free fatty acids, chemokines, interleukins, etc. Studies have shown that decreased expression of mitochondrial stress genes in EAT in patients with coronary heart disease may aggravate atherosclerosis (8). Due to the lack of fascia segmentation, EAT shares the same blood circulation and microcirculation with myocardium (9). Therefore, increased EAT mass causes a high risk of cardiovascular disease and other metabolic syndrome (10). Several studies suggest that the mass of EAT is higher in diabetic patients than in non-diabetic patients, in spite of type 1 DM or type 2 DM, BMI, and total body fat (11, 12). Due to its unique anatomical location and biological activity, EAT can be an important risk factor involved in the development of diabetic cardiovascular disease and leads to death (13). Therefore, modification of EAT may be a therapeutic target to reduce cardiovascular load in diabetic patients.

With the fast development of high throughput technologies, the study of differences in gene expression profiles between diseases and controls through gene microarrays or sequencing technologies has become a powerful tool for screening pathogenic genes and finding therapeutic targets (14). Bioinformatics technology can integrate and analyze huge amounts of molecular biology data to screen out changes in gene expression, biological processes, and protein levels during the development of diseases (15) thus becoming a significant tool in the diagnosis and treatment of clinical diseases. In this study, we obtained two transcriptome expression datasets GSE108971 and GSE179455 from Gene Expression Omnibus (GEO) and deeply analyzed the functions of DEGs (differentially expressed genes), related pathways, core modules, immune infiltration, and potential transcription factors (TFs), aiming to provide new targets for the diagnosis and treatment of diabetes, as well as reduce potential cardiovascular complications in diabetic patients through EAT modification.

## Materials and methods

### Original data acquisition

Transcriptome profiles, namely, GSE108971 and GSE179455, were obtained from the National Center for Biotechnology Information (NCBI) GEO database (16). The RNA expression of GSE108971 was assayed on GPL11154, and GSE179455 was assayed on GPL24676. All participants had severe or non-severe coronary artery disease and underwent elective open-chest coronary artery bypass grafting (CABG), heart valve replacement (VR), or combined (CABG/VR). Participants were divided into two groups based on whether they had T2DM. EAT was collected over the body of the right ventricle or proximal right coronary artery. The GSE108971 dataset consisted of 8 EAT samples from 5 diabetic and 3 non-diabetic human patients. The GSE179455 dataset included 12 EAT samples from 5 diabetic participants and 7 samples from controls.

### Data pre-processing by DESeq2

First, raw counts of the two datasets were annotated with official gene symbols using the annotations file from the Ensembl database. The count files were merged into one file through a Perl script. DESeq2, designed for count data, provides methods to test for differential expression by the use of negative binomial generalized linear models (17). The estimates of dispersion and logarithmic fold changes incorporate data-driven prior distributions. Here, we used the R software (version 4.1.2^1^) package “DESeq2” to obtain DEGs between diabetic and non-diabetic groups. Then, we performed minimal prefiltering to keep only rows that had at least 10 reads in total. We used the DESeqDataSetFromMatrix function to construct the input matrix. Subsequently, DESeq2 estimated the size factors of each gene to normalize and batch correct the sequencing depth and RNA composition. Next, we adjusted the variance by applying a variance stabilizing transformation (VST) to the normalized count to improve PCA visualization and hierarchical clustering. The PCA plot showed that the intracluster difference of a single sample GSM5418471 from GSE179455 was many times larger than the intercluster differences in the data (Supplementary Figure 1). Therefore, we removed this single outlier and reran the DESeq2 analysis. We also used the limma package to visualize the effect of batch-effect removal. Details for data processing, Perl script, and R analysis scripts are shown in Supplementary Datasheet 1.

### Identification of differentially expressed genes

The DEGs between diabetic and non-diabetic samples were identified using the “DESeq2” package in R using an expression count profile. *P*-value < 0.01 and | log_2_FC| > 1 were considered statistically significant. Visualization of DEGs was realized by a volcano map and heatmap using the R packages “ggpubr” (18) and “pheatmap.”

### Gene ontology and Kyoto Encyclopedia of Genes and Genomes pathway enrichment analysis of differentially expressed genes

The Database for Annotation, Visualization, and Integrated Discovery (DAVID^2^) is a powerful online tool to provide systematic and comprehensive functional annotation information for the large-scale gene or protein lists (19). In this study, Gene Ontology (GO) terms (including biological process, molecular function, and cellular component) and Kyoto Encyclopedia of Genes and Genomes (KEGG) pathway enrichment were analyzed using DAVID (version 6.8). The threshold was set at a *P*-value < 0.05.

### Establishment and analysis of protein–protein interaction network

The Search Tool for the Retrieval of Interacting Genes (STRING^3^) is an online database of known and predicted protein–protein interactions (20). In this study, the protein–protein interaction (PPI) network of the above-mentioned DEGs was constructed using STRING (version 4.8), with a threshold of medium confidence ≥ 0.4. Cytoscape (version 3.9.1) is an open-source software project for integrating and visualizing molecular interaction networks (21). CytoHubba (version 0.1), a plug-in for Cytoscape, predicts and explores important nodes and subnetworks using 11 topological algorithms (22). Finally, 10 genes of the PPI network were selected as hub genes according to the degree score through MCC algorithms. Then, GO term analysis of these hub genes was carried out using DAVID. In addition, two significant modules were identified based on the molecular complex detection (MCODE) plug-in, an algorithm that detects densely connected regions in large PPI networks (23). The inclusion criteria were degree cutoff = 2, node score cutoff = 0.2, K-core = 2, and maximum depth = 100.

### Immune cell infiltration analysis

CIBERSORT, an online analytic tool from the Alizadeh Lab and Newman Lab, estimates the abundances of immune cell types (24) using gene expression data. The above-mentioned gene expression normalized profile was uploaded to CIBERSORT, and the percentages of 22 kinds of immune cells (mainly B cells, T cells, NK cells, monocytes, and macrophages) were analyzed using the deconvolution algorithm. Then, bar plots, heatmaps, co-heatmaps, and violin plots were used to visualize the immune infiltration results through R.

### Transcription factors prediction and analysis

Transcription factors are a class of protein molecules that perform the function of regulating gene expression by recognizing specific DNA sequences. ChEA3^4^ is a web-based transcription factor enrichment analysis (TFEA) tool that ranks TFs associated with submitted gene lists (25). It assembles ENCODE, ReMap, and some independently published CHIP-seq data and also integrates transcription factor co-expression data within RNA-seq data from GTEx, TCGA, and ARCHS4. In this study, the DEGs list was submitted to ChEA3, and the top 5 TFs were identified.

## Results

### Preprocessing of datasets

**FIGURE 1:**
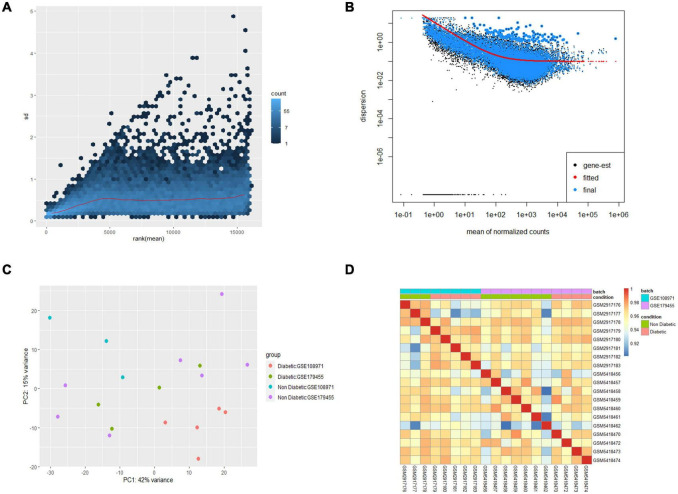
Preprocessing of the two datasets. **(A)** Effects of transformation on the variance. **(B)** Dispersion plot. **(C)** PCA plot after batch corrected. **(D)** Cluster dendrogram of 19 samples.

In this study, 9 EAT samples of diabetic patients and 10 samples of non-diabetic patients from GSE108971 and GSE179455 were finally included and analyzed. The RNA expression raw counts were normalized and batch corrected. Figure 1A shows that the standard deviation is roughly constant along the whole dynamic range for the variance stabilized data. The dispersion plot in Figure 1B shows the final estimates shrunk from the gene-wise estimates toward the fitted estimates. The cluster dendrogram in Figure 1D shows that the overall correlation between samples was high (>0.9), indicating that there were no other outlier samples. Supplementary Figure 2 shows that samples from two batches were separated. While the limma package visualized the removal of batch-effect, the samples were clustered together after batch correction (Figure 1C). In summary, these plots elaborated that our data were of good quality and that differential expression analysis could be performed after preprocessing.

### Identification of differentially expressed genes in diabetic epicardial adipose tissue

**FIGURE 2:**
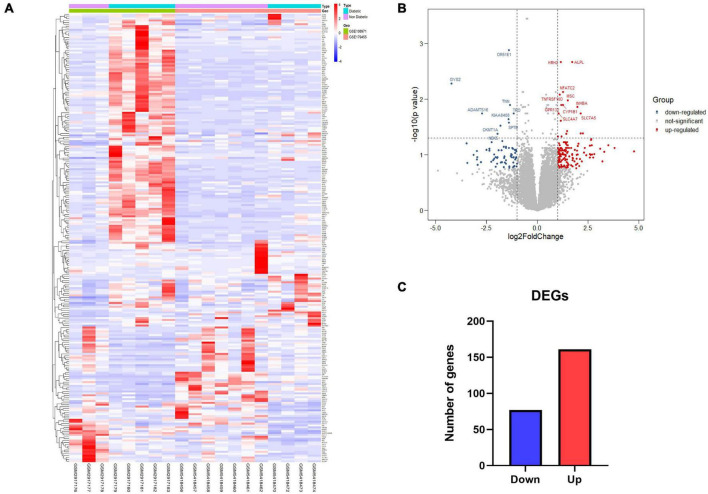
Identification of all differentially expressed genes (DEGs) between diabetic and non-diabetic EAT. **(A)** The heatmap of DEGs. Red represents upregulated genes; blue represents downregulated genes. **(B)** The volcano plot of 238 DEGs. Upregulated genes were in red dots; downregulated genes were in blue dots. *P*-value < 0.01 and | log_2_FC| > 1. **(C)** Numbers of DEGs. 161 genes were upregulated; 77 genes were downregulated. DEGs, differentially expressed genes; EAT, epicardial adipose tissue.

The inclusion criteria for DEGs were *P*-value < 0.01 and | log_2_FC| > 1. Then, the differential expression analysis was performed using DESeq2. Heatmap visualized significant differences in gene expression between diabetic and non-diabetic EAT (Figure 2A). Finally, a total of 238 DEGs were identified, including 161 upregulated genes and 77 downregulated genes (Figures 2B,C).

### Gene ontology and Kyoto Encyclopedia of Genes and Genomes enrichment analysis of differentially expressed genes

**FIGURE 3:**
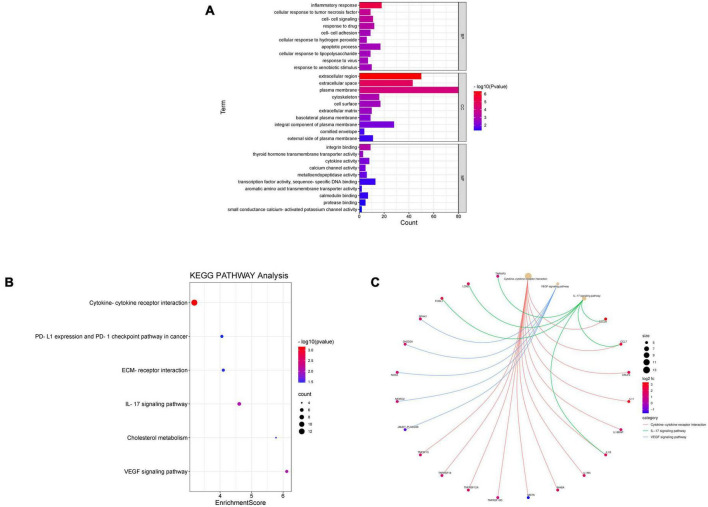
Gene Ontology and KEGG pathway enrichment analysis of DEGs between diabetic and non-diabetic EAT. **(A)** The significant GO terms (biological process, cellular component, and molecular function) of 238 DEGs. **(B)** The significant KEGG pathway analysis of DEGs. *P*-value < 0.05. GO, Gene Ontology; KEGG, Kyoto Encyclopedia of Genes and Genomes. **(C)** Functional pathway analysis of corresponding genes and the top 3 related pathways.

Database for Annotation, Visualization, and Integrated Discovery was used to identify enriched biological themes (GO terms and KEGG pathways). *P*-value < 0.05 was regarded as statistically significant. GO analysis results showed that changes in biological processes (BP) were mainly enriched in the inflammatory response and cellular response to tumor necrosis factor (Figure 3A). Changes in cell components (CC) were mainly enriched in the extracellular region and extracellular space. Changes in molecular function (MF) were mainly enriched in integrin binding and cytokine activity. More details of GO analysis results are shown in Table 1. KEGG pathway analysis revealed that DEGs were mainly enriched in cytokine–cytokine receptor interaction, IL-17 signaling pathway, and VEGF signaling pathway (Figure 3B and Table 2). The top 3 functional pathway analyses mapped the corresponding genes are shown in Figure 3C.

**TABLE 1:** Gene Ontology (GO) functional enrichment analysis of differentially expressed genes (DEGs).

Term	Count	*P*-value
**Biological process**		
GO:0006954∼inflammatory response	18	5.07E-06
GO:0071356∼cellular response to tumor necrosis factor	9	2.35E-04
GO:0007267∼cell-cell signaling	11	3.13E-04
GO:0042493∼response to drug	12	5.54E-04
GO:0098609∼cell-cell adhesion	9	0.001525
GO:0070301∼cellular response to hydrogen peroxide	6	0.001549
GO:0006915∼apoptotic process	17	0.001631
GO:0071222∼cellular response to lipopolysaccharide	9	0.001674
GO:0009615∼response to virus	7	0.001697
GO:0009410∼response to xenobiotic stimulus	10	0.00181
**Cellular component**		
GO:0005576∼extracellular region	50	4.54E-07
GO:0005615∼extracellular space	43	1.58E-05
GO:0005886∼plasma membrane	83	6.84E-05
GO:0005856∼cytoskeleton	16	0.00104
GO:0009986∼cell surface	17	0.002009
GO:0031012∼extracellular matrix	10	0.002436
GO:0016323∼basolateral plasma membrane	9	0.005082
GO:0005887∼integral component of plasma membrane	28	0.00884
GO:0001533∼cornified envelope	4	0.027448
GO:0009897∼external side of plasma membrane	11	0.03066
**Molecular function**		
GO:0005178∼integrin binding	9	6.84E-04
GO:0015349∼thyroid hormone transmembrane transporter activity	3	2.83E-03
GO:0005125∼cytokine activity	8	8.10E-03
GO:0005262∼calcium channel activity	5	9.58E-03
GO:0004222∼metalloendopeptidase activity	6	0.014207
GO:0003700∼transcription factor activity, sequence-specific DNA binding	13	0.032941
GO:0015173∼aromatic amino acid transmembrane transporter activity	2	0.035146
GO:0005516∼calmodulin binding	7	0.035777
GO:0002020∼protease binding	5	0.040826
GO:0016286∼small conductance calcium-activated potassium channel activity	2	0.046586

**TABLE 2:** The Kyoto Encyclopedia of Genes and Genomes (KEGG) pathway analysis of DEGs.

Term	Count	Genes	*P*-value
hsa04060: Cytokine-cytokine receptor interaction	13	IL11, IL1RN, TNFRSF12A, MSTN, TNFSF15, CCL20, TNFRSF18, INHBA, TNFRSF10D, IL18RAP, CCL7, IL1B, CRLF2	6.96E-04
hsa04370: VEGF signaling pathway	5	NOS3, SH2D2A, JMJD7-PLA2G4B, SPHK1, NFATC2	0.008589
hsa04657: IL-17 signaling pathway	6	FOSL1, CCL7, CCL20, IL1B, LCN2, TNFAIP3	0.009305
hsa04979: Cholesterol metabolism	4	ABCG8, LIPG, APOA1, APOB	0.030981
hsa04512: ECM-receptor interaction	5	TNN, ITGA8, SPP1, THBS1, GP5	0.032578
hsa05235: PD-L1 expression and PD-1 checkpoint pathway in cancer	5	MAP2K3, CD274, NFATC2, PDCD1, BATF	0.033761

### Protein–protein interaction network construction and hub genes recognition

**FIGURE 4:**
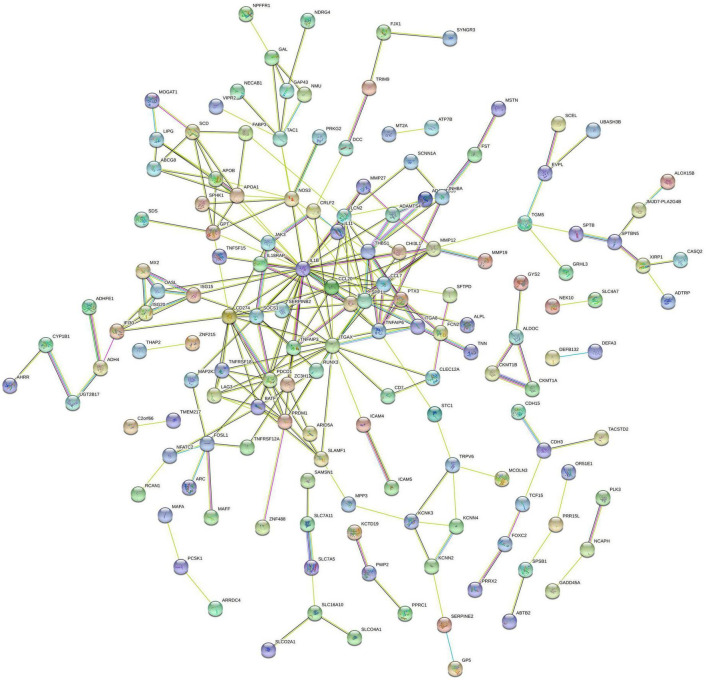
Protein–protein interaction network of proteins constructed by the DEGs. The network included 149 nodes and 254 edges. Medium confidence = 0.40; PPI, protein–protein interaction.

**FIGURE 5:**
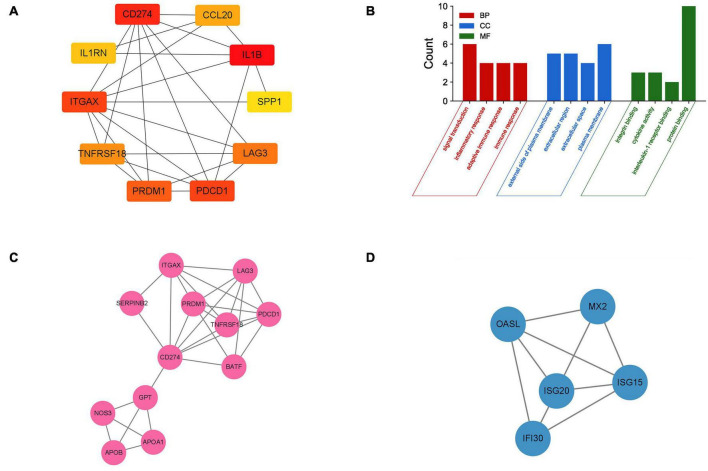
Identification of hub genes and two core modules. **(A)** The top 10 most significant hub genes identified using the plugin cytoHubba. **(B)** GO analysis of 10 hub genes. **(C,D)** Two key modules identified by MODE.

The PPI network of DEGs was constructed using STRING with a medium confidence ≥ 0.4 and visualized using Cytoscape (Figure 4). In total, 149 nodes and 254 edges were involved in this PPI network. Then, 10 genes (IL1B, CD274, PDCD1, ITGAX, PRDM1, LAG3, TNFRSF18, CCL20, IL1RN, and SPP1) were selected as hub genes using plugin cytoHubba (Figures 5A,B). The abbreviations, full names, and detailed functions of hub genes are shown in Table 3. We also identified two core modules using MCODE (Figures 5C,D).

**TABLE 3:** Functions of 10 hub genes.

Gene symbol	Official full name	Function
IL-1β	Interleukin 1 beta	The protein encoded by this gene is a member of the interleukin 1 cytokine family. This cytokine is an important mediator of the inflammatory response, and is involved in a variety of cellular activities, including cell proliferation, differentiation, and apoptosis. Patients with severe Coronavirus Disease 2019 (COVID-19) present elevated levels of pro-inflammatory cytokines such as IL-1B in bronchial alveolar lavage fluid samples. The lung damage induced by the SARS-CoV-2 is to a large extent, a result of the inflammatory response promoted by cytokines such as IL-1β.
CD274	CD274 molecule	This gene encodes an immune inhibitory receptor ligand that is expressed by hematopoietic and non-hematopoietic cells, such as T cells and B cells and various types of tumor cells. Interaction of this ligand with its receptor inhibits T-cell activation and cytokine production. During infection or inflammation of normal tissue, this interaction is important for preventing autoimmunity by maintaining homeostasis of the immune response. In tumor microenvironments, this interaction provides an immune escape for tumor cells through cytotoxic T-cell inactivation.
PDCD1	Programmed Cell Death 1	PDCD1 is an immune-inhibitory receptor expressed in activated T cells; it is involved in the regulation of T-cell functions, including those of effector CD8 + T cells. PDCD1 is expressed in many types of tumors including melanomas, and has demonstrated to play a role in anti-tumor immunity. Moreover, this protein has been shown to be involved in safeguarding against autoimmunity, however, it can also contribute to the inhibition of effective anti-tumor and anti-microbial immunity.
ITGAX	Integrin Subunit Alpha X	This gene encodes the integrin alpha X chain protein. This protein combines with the beta 2 chain (ITGB2) to form a leukocyte-specific integrin referred to as inactivated-C3b (iC3b) receptor 4 (CR4). The alpha X beta 2 complex seems to overlap the properties of the alpha M beta 2 integrin in the adherence of neutrophils and monocytes to stimulated endothelium cells, and in the phagocytosis of complement coated particles.
PRDM1	PR/SET Domain 1	This gene encodes a protein that acts as a repressor of beta-interferon gene expression. Transcription factor that mediates a transcriptional program in various innate and adaptive immune tissue-resident lymphocyte T cell types such as tissue-resident memory T, natural killer and natural killer T cells and negatively regulates gene expression of proteins that promote the egress of tissue-resident T-cell populations from non-lymphoid organs.
LAG3	Lymphocyte Activating 3	LAG3 protein: Inhibitory receptor on antigen activated T-cells. Following TCR engagement, LAG3 associates with CD3-TCR in the immunological synapse and directly inhibits T-cell activation (By similarity). May inhibit antigen-specific T-cell activation in synergy with PDCD1/PD-1, possibly by acting as a coreceptor for PDCD1/PD-1 (By similarity).
TNFRSF18	TNF Receptor Superfamily Member 18	This gene encodes a member of the TNF-receptor superfamily. The encoded receptor has been shown to have increased expression upon T-cell activation, and it is thought to play a key role in dominant immunological self-tolerance maintained by CD25(+) CD4(+) regulatory T cells. Knockout studies in mice also suggest the role of this receptor is in the regulation of CD3-driven T-cell activation and programmed cell death.
CCL20	C-C Motif Chemokine Ligand 20	This antimicrobial gene belongs to the subfamily of small cytokine CC genes. Cytokines are a family of secreted proteins involved in immunoregulatory and inflammatory processes. The CC cytokines are proteins characterized by two adjacent cysteines. The protein encoded by this gene displays chemotactic activity for lymphocytes and can repress proliferation of myeloid progenitors.
IL1RN	Interleukin 1 Receptor Antagonist	The protein encoded by this gene is a member of the interleukin 1 cytokine family. This protein inhibits the activities of interleukin 1, alpha (IL1A) and interleukin 1, beta (IL1B), and modulates a variety of interleukin 1 related immune and inflammatory responses, particularly in the acute phase of infection and inflammation.
SPP1	Secreted Phosphoprotein 1	The protein encoded by this gene is involved in the attachment of osteoclasts to the mineralized bone matrix. The encoded protein is secreted and binds hydroxyapatite with high affinity. The osteoclast vitronectin receptor is found in the cell membrane and may be involved in the binding to this protein. Among its related pathways are FGF signaling pathway and Toll-Like receptor Signaling Pathways. Gene Ontology (GO) annotations related to this gene include cytokine activity and extracellular matrix binding.

### Immune cell infiltration analysis

**FIGURE 6:**
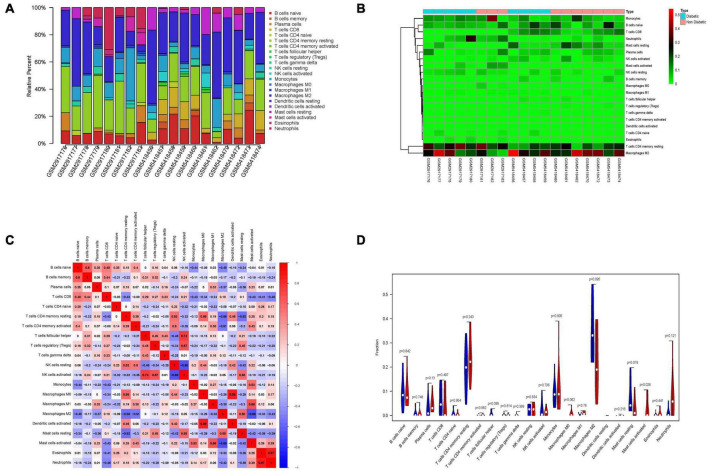
Immune cells infiltration analysis of DEGs between diabetic and non-diabetic EAT using CIBERSORT. **(A)** The relative percentage of 21 immune cell types. **(B)** The heatmap of 21 subgroups of immune cells. **(C)** The correlation analysis among 21 immune cell types. **(D)** The difference in immune infiltration between diabetic and non-diabetic EAT samples. Blue columns represent non-diabetic group; red columns represent the diabetic group.

Immune cell infiltrate deconvolution was obtained from 9 diabetic and 10 non-diabetic EAT using CIBERSORT analysis. Among 22 immune cell types, 21 kinds were detected in one or more patients, except for dendritic cells resting (Figures 6A,B). Interestingly, macrophage M2 and T cells CD4 memory resting made up the largest proportion of these immune cells. Correlation analysis among 20 immune cell types revealed that the activated dendritic cells and macrophage M0 had the strongest positive correlation (γ = 0.88), while the resting and activated NK cells had the strongest negative correlation (γ = –0.68) (Figure 6C). Additionally, the analysis of the ratio in all types of immune cells showed that the number of mast cells activated in diabetic EAT was significantly higher than that in non-diabetic EAT (*P* < 0.05) (Figure 6D).

### Transcription factors prediction and analysis

**FIGURE 7:**
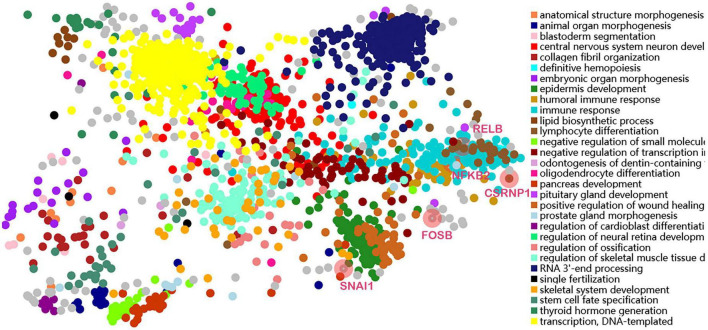
Transcription factors-DEGs coregulatory networks and GO analysis.

Transcription factors were predicted using the online TFEA tool ChEA3. The DEGs list was submitted to the web page, and then 5 potential TFs were obtained according to mean rank by combining multiple databases (Table 4). The smaller the mean rank, the higher the certainty of prediction. The top 5 predicted TFs were CSRNP1, RELB, NFKB2, SNAI1, and FOSB. The TFs-DEGs regulatory network and GO analysis are shown in Figure 7.

**TABLE 4:** The top 5 potential transcription factors of DEGs.

Rank	TFs	Score	Overlapping genes
1	CSRNP1	10.5	23
2	RELB	10.5	54
3	NFKB2	13.0	55
4	SNAI1	14.0	39
5	FOSB	15.0	40

## Discussion

Diabetes mellitus is a group of metabolic diseases characterized by a chronic increase in blood glucose levels. Currently, the prevalence and incidence of diabetes mellitus are rising dramatically worldwide and have become an important cause of cardiovascular disease (26). Visceral obesity is associated with metabolic disorders, including insulin resistance, impaired glucose tolerance, T2DM, and polycystic ovary syndrome (27). The role of visceral fat in the development and progression of T2DM has been extensively studied. Recently, the relationship between EAT and diabetes has attracted a great deal of interest. EAT is the visceral adipose tissue located between the myocardium and epicardium. EAT is an active endocrine organ, capable of secreting many cytokines, growth factors, vasoactive factors, anti-inflammatory, and pro-inflammatory factors (28), and its thickness can be measured using echocardiography, cardiac multidetector computed tomography (CT), and cardiac magnetic resonance imaging (MRI) (29). In patients with heart failure, increased UPR and autophagy in EAT compared to subcutaneous adipose tissue (SAT) are expected to be an early biomarker for identifying cardiomyopathy and a new therapeutic target (30).

Studies have shown that EAT thickness has the potential to predict glucose abnormalities, with significantly increased EAT thickness in patients with insulin resistance and impaired glucose tolerance (31). EAT thickness is higher in type 2 diabetic patients with subclinical atherosclerosis (32). Moreover, increased trans and bound fatty acids in EAT may contribute to the development and progression of atherosclerosis in diabetic patients (33). Notably, it has been shown that EAT overexpresses lipoprotein receptors, such as low-density lipoprotein receptor 1 and very low-density lipoprotein receptor, playing a role in the changes in lipid metabolism normally associated with type 2 diabetes (34). In general, EAT mass and thickness are significantly increased in diabetic patients, and EAT could be a new independent predictor of diabetes as well as a new target for diabetes drug therapy (35), providing new horizons for clinical treatment. Moreover, EAT lacks tissue demarcation with the myocardium and shares an unobstructed microcirculation, thus becoming sensors for adverse systemic inflammation and metabolic disorders in the heart (36). Thus, it is of great significance to explore the difference in gene expression between diabetic and non-diabetic EAT.

In this study, bioinformatics analysis was used to determine the transcriptional effect of T2DM on EAT. Our results showed that the gene expression of EAT in diabetic patients is significantly different from that of non-diabetic patients. A total of 238 DEGs were identified with 161 genes upregulated and 77 genes downregulated. Genes associated with inflammation and cytokines are significantly altered in diabetic EAT.

Gene ontology analysis demonstrated that DEGs were mainly enriched in the inflammatory response, cellular response to tumor necrosis factor, cytokine activity, and cell-cell signaling. KEGG pathway analysis showed that cytokine–cytokine receptor interaction, IL-17 signaling pathway, and VEGF signaling pathway were highly enriched in the diabetic EAT samples. Inflammation is thought to contribute to the development and maintenance of many chronic diseases, including atherosclerosis and diabetes (37). Previous studies have shown that EAT is a source of multiple inflammatory mediators and cytokines in patients at high risk for heart disease (38, 39). Therefore, diabetes may activate genes in different pathways of EAT to respond to inflammation and accelerate the progression of cardiovascular disease.

Then, the PPI network of DEGs was constructed; IL-1β, CD274, PDCD1, ITGAX, PRDM1, LAG3, TNFRSF18, CCL20, IL1RN, and SPP1 were selected as hub genes. We speculate that these differential molecules in EAT may be involved in the pathophysiological processes of diabetic cardiovascular complications.

The most significant gene in the diabetic EAT was IL-1β. IL-1β is an important mediator of the inflammatory response, participating in cell proliferation, differentiation, and apoptosis. IL-1β is crucially involved in the pathogenesis of coronary atherosclerotic diseases (CAD) (40). Anti-inflammatory therapy targeting IL-1β reduced cardiovascular events in a randomized trial (41). In addition, regional IL-1β in EAT was an independent risk factor for persistent atrial fibrillation (42). Therefore, IL-1β in EAT might be a target to reduce cardiovascular inflammation and complication. CD274 and its ligands (PD-L1 and PD-L2) deliver inhibitory signals that regulate the balance between T-cell activation, tolerance, and immunopathology (43). It is critical for cancer immune evasion and thus has become one of the major targets in anticancer immunotherapy (44). T2DM is a kind of chronic metabolic disease and can alter the immune status of EAT. CD274 may become an immunotherapy target in diabetes-related cardiovascular complications. PDCD1 is a ligand of CD274 and is involved in safeguarding against autoimmunity (45). In addition, LAG3 may inhibit antigen-specific T-cell activation in synergy with PDCD1/PD-1, possibly by acting as a coreceptor for PDCD1/PD-1 (by similarity). ITGAX encodes the integrin alpha X-chain protein. However, little is known about the role of ITGAX in EAT, and further research and exploration are needed in the future. PRDM1 encodes a protein that acts as a repressor of ILβ gene expression. We speculate that PRDM1 may participate in the autoimmunity response in diabetic EAT. TNFRSF18 encodes a member of the TNF-receptor superfamily. TNF has been identified as a key regulator of the inflammatory response (46). CCL20 belongs to the subfamily of small cytokine CC genes. Cytokines are a family of secreted proteins involved in the immunoregulatory and inflammatory processes. An observational study showed that CCL20 had a strong association with vascular endothelial inflammation, reflected systemic inflammation (47), and thus may become a potential biomarker of impaired vascular function in diabetes. Moreover, the CCR6-CCL20 inhibitor has been identified as possessing a high medicinal potential to treat autoimmune and inflammatory diseases (48). The protein encoded by IL1RN is a member of the IL-1 cytokine family. It inhibits the activities of IL-α and IL-1β and modulates a variety of IL-1-related immune and inflammatory responses. SPP1 is involved in the attachment of osteoclasts to the mineralized bone matrix. Its encoded protein is also a cytokine that upregulates the expression of IFN-γ and IL-12. SPP1 may play an important role in acute myocardial infarction after ischemia and reperfusion injury (49). Moreover, SPP1 promotes human cardiac fibroblast fibrosis by reducing p27 expression through the regulation of the PI3K/Akt signaling pathway (50). We speculate that SPP1 may be involved in inflammatory phenotypic alterations in the diabetic EAT by interacting with cytokines.

In addition, our study identified a number of transcription factors of 238 DEGs. The top 5 were CSRNP1, RELB, NFKB2, SNAI1, and FOSB. CSRNP1 encodes a protein that localizes to the nucleus. Expression of this gene is induced in response to the elevated levels of axin and has the function of a tumor suppressor. RELB, also known as the NF-κB subunit, is a pleiotropic transcription factor. Also, NFKB2 encodes a subunit of the transcription factor complex nuclear factor NFκB. The NFκB complex is expressed in numerous cell types and functions as a central activator of genes involved in inflammation and immune function (51). SNAI1 is a zinc-finger transcriptional repressor involved in the induction of the epithelial to mesenchymal transition (EMT), formation and maintenance of embryonic mesoderm, growth arrest, survival, and cell migration (52). The FOS gene family contains 4 members: FOS, FOSB, FOSL1, and FOSL2. The FOS proteins have been implicated as regulators of cell proliferation, differentiation, and transformation (53). A study showed that SIRT3 could mediate the intricate profibrotic and proinflammatory responses of cardiac cells through the modulation of the FOS pathway (54).

We also analyzed the immune infiltration in the EAT microenvironment using CIBERSORT. Many studies have been performed showing increased EAT in diabetic patients, but in-depth studies on immune cell types and inflammatory mediator components are lacking. Our analysis found that there was little difference in the proportion of various types of immune cells between diabetic and non-diabetic EAT. This may be attributed to two reasons. First, all patients included in the study had coronary heart disease or valvular heart disease and underwent elective open-heart surgery, which may have triggered the immune response of the organism. Second, almost all of the diabetic patients included in the study were taking the glucose-lowering drug metformin, thus suppressing the immune and inflammatory responses in the body (55). Thus, diabetes appears to alter certain inflammatory mediators and cytokines of EAT rather than generate an effect on the immune phenotype. In addition, macrophage M2 and T cells CD4 resting are the primary immune cell types present in EAT. The anatomical location of EAT is extremely close to the coronary arteries and myocardium, so the vasoactive molecules, adipokines, and growth factors it synthesizes are directly involved in the development of cardiovascular disease through paracrine and vascular secretion (56). A study using Bulk RNA-seq revealed that genes related to lipid metabolism (GYS2, GPAT3, CRAT, FASN, ACADVL, DGAT1, DGAT2, NAT8L, and SCD) and genes related to adipogenesis (HES1, MXD3, NR4A2, RGS2, PPP1R15B, ADAMTS1, CEBPD, and DM7A) were significantly altered in the EAT of diabetic patients. The levels of immune mediators (TNF-α, IFN-γ, IL-1, IL-6, leptin, etc.) were also extremely elevated (57). Previous studies have found increased secretion of leptin and IL-6 and decreased secretion of cardioprotective lipocalin in EAT in patients with coronary artery disease, leading to immune cell activation and inflammatory responses (58). Diabetes alters fatty acid composition in EAT, and glucose uptake and lipid metabolism are impaired in EAT in heart failure patients with diabetes (59). This is in line with the direction of our findings. We venture to speculate that the above-mentioned molecules may serve as modification targets for EAT to mitigate the risk of cardiovascular complications by improving the inflammatory and lipid metabolic status of diabetic patients. In the future, more research is needed on the inflammatory infiltration and immune cell microenvironment of EAT in diabetic patients.

### Limitations

Our study first reported differences in gene expression of EAT between diabetic and non-diabetic patients, but some limitations still remain. First, the small sample size may have biased the analysis results. Second, EAT can only be sampled during cardiac surgery and cannot be collected from healthy volunteers. Thus, it is difficult to obtain EAT tissues from humans for experimental validation at the molecular level due to ethical and moral restrictions.

## Conclusion

In this study, comprehensive bioinformatic analysis was used to identify the differences in EAT between diabetic and non-diabetic patients. IL-1β, CD274, PDCD1, ITGAX, PRDM1, LAG3, TNFRSF18, CCL20, IL1RN, and SPP1 were mined as hub genes; related pathways included a response to inflammation response and cytokine–cytokine receptor interaction. CSRNP1, RELB, NFKB2, SNAI1, and FOSB were regarded as key transcription factors. Moreover, the immune cells (including macrophage M2, B cell naïve, T cells CD8, and mast cells activated) may participate in inflammatory and metabolic alterations of EAT in diabetic patients. Diabetes mainly alters the inflammatory response and cytokine activity of EAT and may have adverse cardiovascular effects through the unique geographic location and secretory function of EAT.

## Data availability statement

The datasets presented in this study can be found in online repositories. The names of the repository/repositories and accession number(s) can be found in the article/Supplementary material.

## Author contributions

HY and BX designed and implemented the subject. TX and DW made substantial contributions to acquisition of data. WY and BL made substantial contributions to analysis and interpretation of data. HY wrote the manuscript. QS gave final approval of the version to be published. All authors read and approved the final manuscript.

## Footnotes

1
https://www.R-project.org/

2
http://David.ncifcrf.gov/home.jsp

3
http://string-db.org

4
https://amp.pharm.mssm.edu/ChEA3

## Supplementary material

SUPPLEMENTARY FIGURE 1 PCA plot before removing outliers.

SUPPLEMENTARY FIGURE 2 PCA plot after removing outliers.

SUPPLEMENTARY DATASHEET 1 Perl script and R analysis scripts of data processing.

## Abbreviations

EAT: epicardial adipose tissue
GEO: Gene Expression Omnibus
DEGs: differentially expressed genes
GO: Gene Ontology
KEGG: Kyoto Encyclopedia of Genes and Genomes
T2DM: type 2 diabetes
PPI: protein–protein interaction
TFs: transcription factors
NCBI: National Center for Biotechnology Information
CABG: coronary artery bypass grafting
VR: valve replacement
TFEA: transcription factor enrichment analysis
BP: biological processes
CC: cell component
MF: molecular function
CT: computed tomography
MRI: magnetic resonance imaging
SAT: subcutaneous adipose tissue
EMT: epithelial to mesenchymal transition.

## References

[1] Zheng Y Ley SH Hu FB. Global aetiology and epidemiology of type 2 diabetes mellitus and its complications. *Nat Rev Endocrinol.* (2018) 14:88–98. 10.1038/nrendo.2017.151 29219149

[2] Khan MAB Hashim MJ King KK Govender RD Mustafa H Al AKaabi J. Epidemiology of type 2 diabetes - global burden of disease and forecasted trends. *J Epidemiol Glob Health.* (2020) 10:107–11. 10.2991/jegh.k.191028.001 32175717 PMC7310804

[3] Glovaci D Fan W Wong ND. Epidemiology of diabetes mellitus and cardiovascular disease. *Curr Cardiol Rep.* (2019) 21:21. 10.1007/s11886-019-1107-y 30828746

[4] Balakumar P Maung UK Jagadeesh G. Prevalence and prevention of cardiovascular disease and diabetes mellitus. *Pharmacol Res.* (2016) 113:600–9. 10.1016/j.phrs.2016.09.040 27697647

[5] Marchington JM Mattacks CA Pond CM. Adipose tissue in the mammalian heart and pericardium: structure, foetal development and biochemical properties. *Comp Biochem Physiol B.* (1989) 94:225–32. 10.1016/0305-0491(89)90337-4 2591189

[6] Zangi L Oliveura MS Ye LY Ma Q Sultana N Hadas Y Insulin-like growth factor 1 receptor-dependent pathway drives epicardial adipose tissue formation after myocardial injury. *Circulation.* (2017) 135:59–72. 10.1161/CIRCULATIONAHA.116.022064 27803039 PMC5195872

[7] Sacks HS Fain JN. Human epicardial adipose tissue: a review. *Am Heart J.* (2007) 153:907–17. 10.1016/j.ahj.2007.03.019 17540190

[8] Kratochvilova H Mráz M Kasperová BJ Hlaváèek D Mahrík J Laòková I Different expression of mitochondrial and endoplasmic reticulum stress genes in epicardial adipose tissue depends on coronary atherosclerosis. *Int J Mol Sci.* (2021) 22:4538. 10.3390/ijms22094538 33926122 PMC8123607

[9] Perez-Miguelsanz J Jiménez-Ortega V Cano-Barquilla P Garaulet M Esquifino AI Varela-Moreiras G Early appearance of epicardial adipose tissue through human development. *Nutrients.* (2021) 13:2906. 10.3390/nu13092906 34578784 PMC8469969

[10] Villasante Fricke AC Iacobellis G. Epicardial adipose tissue: clinical biomarker of cardio-metabolic risk. *Int J Mol Sci.* (2019) 20:5989. 10.3390/ijms20235989 31795098 PMC6929015

[11] Kleinaki Z Agouridis AP Zafeiri M Xanthos T Tsioutis C. Epicardial adipose tissue deposition in patients with diabetes and renal impairment: analysis of the literature. *World J Diabetes.* (2020) 11:33–41. 10.4239/wjd.v11.i2.33 32064034 PMC6969709

[12] Li Y Liu B Li Y Jing X Den S Yan Y Epicardial fat tissue in patients with diabetes mellitus: a systematic review and meta-analysis. *Cardiovasc Diabetol.* (2019) 18:3. 10.1186/s12933-019-0807-3 30630489 PMC6327515

[13] Christensen RH von Scholten BJ Lehrskov LL Rossing P Jorgensen PG. Epicardial adipose tissue: an emerging biomarker of cardiovascular complications in type 2 diabetes? *Ther Adv Endocrinol Metab.* (2020) 11:2042018820928824. 10.1177/2042018820928824 32518616 PMC7252363

[14] Kim K Zakharkin SO Allison DB. Expectations, validity, and reality in gene expression profiling. *J Clin Epidemiol.* (2010) 63:950–9. 10.1016/j.jclinepi.2010.02.018 20579843 PMC2910173

[15] Ouzounis CA Valencia A. Early bioinformatics: the birth of a discipline–a personal view. *Bioinformatics.* (2003) 19:2176–90. 10.1093/bioinformatics/btg309 14630646

[16] Barrett T Wilhite ST Ledoux P Evangelista C Kim IF Tomashevsky M NCBI GEO: archive for functional genomics data sets–update. *Nucleic Acids Res.* (2013) 41:D991–5. 10.1093/nar/gks1193 23193258 PMC3531084

[17] Love MI Huber W Anders S. Moderated estimation of fold change and dispersion for RNA-seq data with DESeq2. *Genome Biol.* (2014) 15:550. 10.1186/s13059-014-0550-8 25516281 PMC4302049

[18] Xia X Li Y. Comprehensive analysis of transcriptome data stemness indices identifies key genes for controlling cancer stem cell characteristics in gastric cancer. *Transl Cancer Res.* (2020) 9:6050–61. 10.21037/tcr-20-704 35117216 PMC8797465

[19] Huang da W Sherman BT Lempicki RA. Systematic and integrative analysis of large gene lists using DAVID bioinformatics resources. *Nat Protoc.* (2009) 4:44–57. 10.1038/nprot.2008.211 19131956

[20] Szklarczyk D Gable AL Nastou KC Lyon D Kirsch R Pyysalo S The STRING database in 2021: customizable protein-protein networks, and functional characterization of user-uploaded gene/measurement sets. *Nucleic Acids Res.* (2021) 49:D605–12. 10.1093/nar/gkaa1074 33237311 PMC7779004

[21] Shannon P Markiel A Ozier O Baliga NS Wang JT Ramage D Cytoscape: a software environment for integrated models of biomolecular interaction networks. *Genome Res.* (2003) 13:2498–504. 10.1101/gr.1239303 14597658 PMC403769

[22] Chin C-H Chen S-H Wu H-H Ho C-W Ko M-T Lin C-Y. Cytohubba: identifying hub objects and sub-networks from complex interactome. *BMC Syst Biol.* (2014) 8(Suppl 4):S11. 10.1186/1752-0509-8-S4-S11 25521941 PMC4290687

[23] Bader GD Hogue CW. An automated method for finding molecular complexes in large protein interaction networks. *BMC Bioinformatics.* (2003) 4:2. 10.1186/1471-2105-4-2 12525261 PMC149346

[24] Chen B Khodadoust MS Liu CL Newman AM Alizadeh AA. Profiling tumor infiltrating immune cells with CIBERSORT. *Methods Mol Biol.* (2018) 1711:243–59. 10.1007/978-1-4939-7493-1_12 29344893 PMC5895181

[25] Keenan AB Torre D Lachmann A Leong AK Wojciechowicz ML Utti V ChEA3: transcription factor enrichment analysis by orthogonal omics integration. *Nucleic Acids Res.* (2019) 47:W212–24. 10.1093/nar/gkz446 31114921 PMC6602523

[26] Dal Canto E Ceriello A Rydén L Ferrini M Hansen TB Schnell O Diabetes as a cardiovascular risk factor: an overview of global trends of macro and micro vascular complications. *Eur J Prev Cardiol.* (2019) 26:25–32. 10.1177/2047487319878371 31722562

[27] Neeland IJ Ross R Després J-P Matsuzawa Y Yamashita S Shai I Visceral and ectopic fat, atherosclerosis, and cardiometabolic disease: a position statement. *Lancet Diabetes Endocrinol.* (2019) 7:715–25. 10.1016/s2213-8587(19)30084-1 31301983

[28] Iacobellis G Corradi D Sharma AM. Epicardial adipose tissue: anatomic, biomolecular and clinical relationships with the heart. *Nat Clin Pract Cardiovasc Med.* (2005) 2:536–43. 10.1038/ncpcardio0319 16186852

[29] Iacobellis G. Epicardial adipose tissue in contemporary cardiology. *Nat Rev Cardiol.* (2022) 19:593–606. 10.1038/s41569-022-00679-9 35296869 PMC8926097

[30] Burgeiro A Fonseca AC Espinoza D Carvalho L Lourenco N Antunes M Proteostasis in epicardial versus subcutaneous adipose tissue in heart failure subjects with and without diabetes. *Biochim Biophys Acta Mol Basis Dis.* (2018) 1864:2183–98. 10.1016/j.bbadis.2018.03.025 29625179 PMC6375688

[31] Iacobellis G Leonetti F. Epicardial adipose tissue and insulin resistance in obese subjects. *J Clin Endocrinol Metab.* (2005) 90:6300–2. 10.1210/jc.2005-1087 16091479

[32] Cetin M Cakici M Polat M Suner A Zencir C Ardic I. Relation of epicardial fat thickness with carotid intima-media thickness in patients with type 2 diabetes mellitus. *Int J Endocrinol.* (2013) 2013:769175. 10.1155/2013/769175 23762053 PMC3665232

[33] Pezeshkian M Mahtabipour MR. Epicardial and subcutaneous adipose tissue Fatty acids profiles in diabetic and non-diabetic patients candidate for coronary artery bypass graft. *Bioimpacts.* (2013) 3:83–9. 10.5681/bi.2013.004 23878791 PMC3713874

[34] Nasarre L Juan-Babot O Gastelurrutia P Llucia-Valldeperas A Badimon L Bayes-Genis A Low density lipoprotein receptor-related protein 1 is upregulated in epicardial fat from type 2 diabetes mellitus patients and correlates with glucose and triglyceride plasma levels. *Acta Diabetol.* (2014) 51:23–30. 10.1007/s00592-012-0436-8 23096408

[35] Iacobellis G. Epicardial adipose tissue in endocrine and metabolic diseases. *Endocrine.* (2014) 46:8–15. 10.1007/s12020-013-0099-4 24272604

[36] Packer M. Epicardial adipose tissue may mediate deleterious effects of obesity and inflammation on the myocardium. *J Am Coll Cardiol.* (2018) 71:2360–72. 10.1016/j.jacc.2018.03.509 29773163

[37] Freitas Lima LC de Andrade Braga V de Franca Silva MDS de Campos Cruz J Sousa Santos SH de Oliveira Monteiro MM Adipokines, diabetes and atherosclerosis: an inflammatory association. *Front Physiol.* (2015) 6:304. 10.3389/fphys.2015.00304 26578976 PMC4630286

[38] Karastergiou K Evans I Ogston N Miheisi N Nair D Kaski J-C Epicardial adipokines in obesity and coronary artery disease induce atherogenic changes in monocytes and endothelial cells. *Arterioscler Thromb Vasc Biol.* (2010) 30:1340–6. 10.1161/ATVBAHA.110.204719 20395594

[39] Mazurek T Zhang L Zalewski A Mannion JD Diehl JT Arafat H Human epicardial adipose tissue is a source of inflammatory mediators. *Circulation.* (2003) 108:2460–6. 10.1161/01.Cir.0000099542.57313.C5 14581396

[40] Parisi V Petraglia L Cabaro S D’Esposito V Bruzzese D Ferraro G Imbalance between interleukin-1β and interleukin-1 receptor antagonist in epicardial adipose tissue is associated with non ST-segment elevation acute coronary syndrome. *Front Physiol.* (2020) 11:42. 10.3389/fphys.2020.00042 32116755 PMC7012938

[41] Kitagawa T Hattori T Sentani K Senoo A Fujii Y Takahashi S Relationship between interleukin-1β gene expression in epicardial adipose tissue and coronary atherosclerosis based on computed tomographic analysis. *J. Cardiovasc Comput Tomogr.* (2021) 15:175–9. 10.1016/j.jcct.2020.06.199 32819873

[42] Liu Q Zhang F Yang M Zhong J. Increasing level of interleukin-1β in epicardial adipose tissue is associated with persistent atrial fibrillation. *J. Interferon Cytokine Res.* (2020) 40:64–9. 10.1089/jir.2019.0098 31584315

[43] Keir ME Butte MJ Freeman GJ Sharpe AH. PD-1 and its ligands in tolerance and immunity. *Annu Rev Immunol.* (2008) 26:677–704. 10.1146/annurev.immunol.26.021607.090331 18173375 PMC10637733

[44] Huang X Zhang Q Lou Y Wang J Zhao X Wang L USP22 deubiquitinates CD274 to suppress anticancer immunity. *Cancer Immunol Res.* (2019) 7:1580–90. 10.1158/2326-6066.Cir-18-0910 31399419

[45] Francisco LM Sage PT Sharpe AH. The PD-1 pathway in tolerance and autoimmunity. *Immunol Rev.* (2010) 236:219–42. 10.1111/j.1600-065X.2010.00923.x 20636820 PMC2919275

[46] Bradley JR. TNF-mediated inflammatory disease. *J Pathol.* (2008) 214:149–60. 10.1002/path.2287 18161752

[47] Elnabawi YA Garshick MS Tawil M Barrett TJ Fisher EA Lo Sicco K CCL20 in psoriasis: a potential biomarker of disease severity, inflammation, and impaired vascular health. *J Am Acad Dermatol.* (2021) 84:913–20. 10.1016/j.jaad.2020.10.094 33259876 PMC8049184

[48] Ranasinghe R Eri R. Modulation of the CCR6-CCL20 axis: a potential therapeutic target in inflammation and cancer. *Medicina.* (2018) 54:88. 10.3390/medicina54050088 30453514 PMC6262638

[49] Li L Huang J Zhao Z Wen Z Li K Ma T Decreased Spp1 expression in acute myocardial infarction after ischemia and reperfusion injury. *Cardiol Res Pract.* (2021) 2021:3925136. 10.1155/2021/3925136 34426769 PMC8380156

[50] Wang X Li H Zhang Z Zhang Y Li Z Wang X Diversity among differentially expressed genes in atrial appendages of atrial fibrillation: the role and mechanism of SPP1 in atrial fibrosis. *Int J Biochem Cell Biol.* (2021) 141:106074. 10.1016/j.biocel.2021.106074 34474184

[51] Millet P McCall C Yoza B. RelB: an outlier in leukocyte biology. *J Leukocyte Biol.* (2013) 94:941–51. 10.1189/jlb.0513305 23922380 PMC3800064

[52] Cho ES Kang HE Kim NH Yook JI. Therapeutic implications of cancer epithelial-mesenchymal transition (EMT). *Arch Pharm Res.* (2019) 42:14–24. 10.1007/s12272-018-01108-7 30649699

[53] Milde-Langosch K. The Fos family of transcription factors and their role in tumourigenesis. *Eur J Cancer.* (2005) 41:2449–61. 10.1016/j.ejca.2005.08.008 16199154

[54] Palomer X Román-Azcona MS Pizarro-Delgado J Planavila A Villarroya F Valensuela-Alcaraz B SIRT3-mediated inhibition of FOS through histone H3 deacetylation prevents cardiac fibrosis and inflammation. *Signal Transduct Target Ther.* (2020) 5:14. 10.1038/s41392-020-0114-1 32296036 PMC7046732

[55] Sanchez-Rangel E Inzucchi SE. Metformin: clinical use in type 2 diabetes. *Diabetologia.* (2017) 60:1586–93. 10.1007/s00125-017-4336-x 28770321

[56] Ansaldo AM Montecucco F Sahebkar A Dallegri F Carbone F. Epicardial adipose tissue and cardiovascular diseases. *Int J Cardiol.* (2019) 278:254–60. 10.1016/j.ijcard.2018.09.089 30297191

[57] Vyas V Blythe H Wood EG Sandhar B Sarker S-J Balmforth D Obesity and diabetes are major risk factors for epicardial adipose tissue inflammation. *JCI Insight.* (2021) 6:e145495. 10.1172/jci.insight.145495 34283808 PMC8409986

[58] Gruzdeva OV Dyleva YA Belik EV Sinitsky MY Stasev AN Kokov AN Relationship between epicardial and coronary adipose tissue and the expression of adiponectin, leptin, and interleukin 6 in patients with coronary artery disease. *J Pers Med.* (2022) 12:129. 10.3390/jpm12020129 35207618 PMC8877574

[59] Burgeiro A Fhurmann A Cherian S Espinoza D Jarak I Carvalho RA Glucose uptake and lipid metabolism are impaired in epicardial adipose tissue from heart failure patients with or without diabetes. *Am J Physiol Endocrinol Metab.* (2016) 310:E550–64. 10.1152/ajpendo.00384.2015 26814014 PMC4824138

