# Supplementary figures and images for: Identification of key genes and mechanisms of epicardial adipose tissue in patients with diabetes through bioinformatic analysis
Source: Front Cardiovasc Med. 2022 Sep 9;9:927397. doi: 10.3389/fcvm.2022.927397 (PMC9500152; doi:10.3389/fcvm.2022.927397)

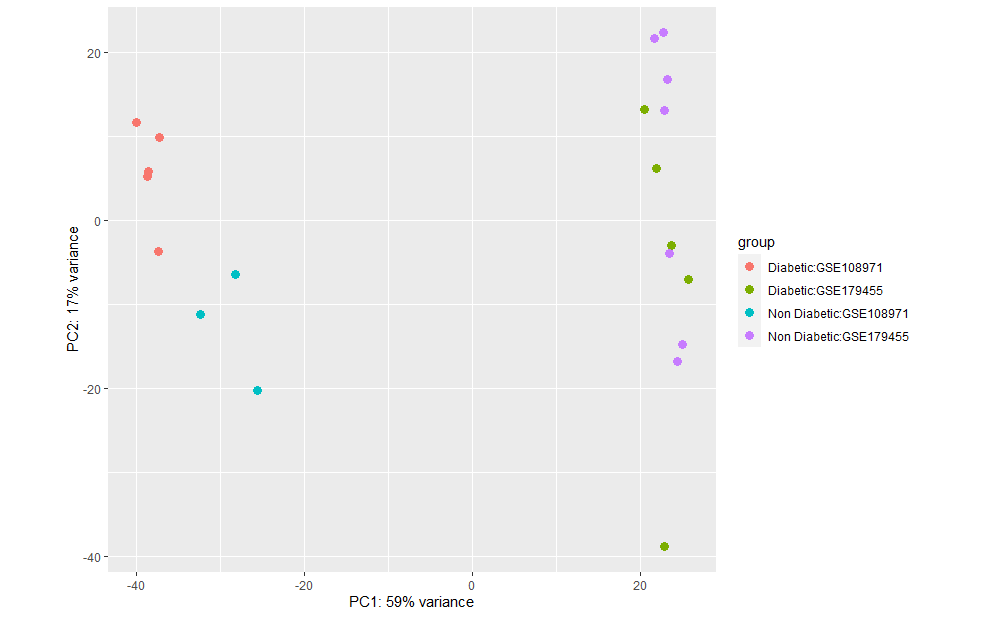

Supplement: SUPPLEMENTARY FIGURE 1 — PCA plot before removing outliers. [file Image_1.TIFF]

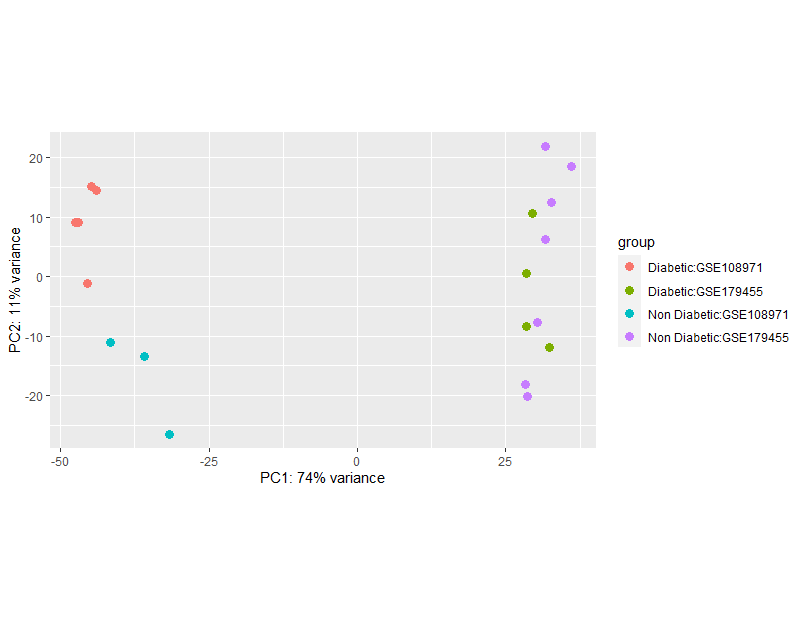

Supplement: SUPPLEMENTARY FIGURE 2 — PCA plot after removing outliers. [file Image_2.TIFF]
