# Supplementary material for: Identification of key genes and mechanisms of epicardial adipose tissue in patients with diabetes through bioinformatic analysis
Source: Front Cardiovasc Med. 2022 Sep 9;9:927397. doi: 10.3389/fcvm.2022.927397 (PMC9500152; doi:10.3389/fcvm.2022.927397)
Supplement: SUPPLEMENTARY DATASHEET 1 — Perl script and R analysis scripts of data processing. [file Data_Sheet_1.DOCX]

###Perl script for merge the two datasets

use strict;

use warnings;

my $file1=$ARGV[0];

my $file2=$ARGV[1];

my $out=$ARGV[2];

my %hash=();

open(RF,"$file1") or die $!;

while(my $line=<RF>){

chomp($line);

my @arr=split(/\t/,$line);

my $gene=shift(@arr);

$hash{$gene}=join("\t",@arr);

}

close(RF);

open(RF,"$file2") or die $!;

open(WF,">$out") or die $!;

while(my $line=<RF>){

chomp($line);

my @arr=split(/\t/,$line);

my $gene=shift(@arr);

if(exists $hash{$gene}){

print WF $gene . "\t" . $hash{$gene} . "\t" . join("\t",@arr) . "\n";

}

}

close(WF);

close(RF);

###Normalization and batch correction

setwd("C:\\Users\\YyyAhead\\Desktop\\Analyse")

mycounts<-read.csv("mycounts.csv",row.names = 1)

head(mycounts)

dim(mycounts)

mycounts1<-mycounts[rowSums(mycounts) >= 10,]

dim(mycounts1)

mymeta<-read.csv("mymeta.csv",stringsAsFactors = T)

mymeta

colnames(mycounts1) == mymeta$sample

library(DESeq2)

## Create DESeq2Dataset object

dds <- DESeqDataSetFromMatrix(countData=mycounts1,

colData=mymeta,

design=~batch+condition)

dds$condition <- factor(dds$condition, levels = c("Non Diabetic","Diabetic"))

## Run analysis

dds <- DESeq(dds)

NormalizedCounts <- counts(dds, normalized=TRUE)

write.table(NormalizedCounts, file="NormalizedCounts.txt",

sep="\t", quote=F, col.names=NA)

res <- results(dds)

res

write.csv(res,file="DiffExp.csv",

quote = F)

### Transform counts for data visualization

vsd <- vst(dds, blind=FALSE)

head(assay(vsd),3)

### Effects of transformation on variance

library("vsn")

meanSdPlot(assay(vsd))

### Dispersion plot

plotDispEsts(dds)

### Plot PCA

plotPCA(vsd, intgroup=c("condition", "batch"))

### Plot PCA after batch removed by limma

mat <- assay(vsd)

mm <- model.matrix(~+condition, colData(vsd))

mat <- limma::removeBatchEffect(mat, batch=vsd$batch, design=mm)

assay(vsd) <- mat

plotPCA(vsd, intgroup=c("condition", "batch"))

### Extract the rlog matrix from the object

vsd_mat <- assay(vsd)

### Compute pairwise correlation values

vsd_cor <- cor(vsd_mat)

head(vsd_cor)

### Load pheatmap package

library(pheatmap)

select <- order(rowMeans(counts(dds,normalized=TRUE)),

decreasing=TRUE)[1:20]

df <- as.data.frame(colData(dds)[,c("condition","batch")])

### Plot heatmap

pheatmap(vsd_cor,cluster_rows=FALSE,

cluster_cols=FALSE,annotation_col=df)

### Pheatmap of 238 DEGs

rt=read.table("NormalizedCounts.txt",sep="\t",header=T,check.names=F)

rt=as.matrix(rt)

rownames(rt)=rt[,1]

exp=rt[,2:ncol(rt)]

dimnames=list(rownames(exp),colnames(exp))

rt=matrix(as.numeric(as.matrix(exp)),nrow=nrow(exp),dimnames=dimnames)

library(pheatmap)

Geo=c(rep("GSE108971",8),rep("GSE179455",11))

Type=c(rep("Non Diabetic",3),rep("Diabetic",5),rep("Non Diabetic",7),rep("Diabetic",4))

names(Geo)=colnames(rt)

ann=cbind(Geo,Type)

ann=as.data.frame(ann)

tiff(file="heatmap.tiff",

width = 35,

height =45,

units ="cm",

compression="lzw",

bg="white",

res=300)

pheatmap(rt, annotation=ann,

color = colorRampPalette(c("blue", "white", "red"))(40),

cluster_cols =F,

scale="row",

fontsize_row=4,

fontsize_col=12)

dev.off()

###Volcano Plot of 238 DEGs

library(ggpubr)

library(ggthemes)

deg.data<-read.table("DiffExp.xls",header=T,sep="\t")

head(deg.data)

deg.data$logP<- -log10(deg.data$padj)

ggscatter(deg.data,x="logFC",y="logP")+theme_base()

deg.data$Group="not-significant"

deg.data$Group[which((deg.data$pvalue<0.01)&(deg.data$logFC>1))]="up-regulated"

deg.data$Group[which((deg.data$pvalue<0.01)&(deg.data$logFC< -1))]="down-regulated"

ggscatter(deg.data,x="logFC",y="logP",color="Group")+theme_base()

ggscatter(deg.data,x="logFC",y="logP",

color="Group",

palette=c("blue","black","red"),size=1)+theme_base()

ggscatter(deg.data,x="logFC",y="logP",

color="Group",

palette=c("blue","black","red"),size=1)+theme_base()+

geom_hline(yintercept=1.30,linetype="dashed")+

geom_vline(xintercept=c(-1,1),linetype="dashed")

deg.data$Label=""

deg.data<-deg.data[order(deg.data$pvalue),]

up.genes<-head(deg.data$id[which(deg.data$Group=="up-regulated")],10)

down.genes<-head(deg.data$id[which(deg.data$Group=="down-regulated")],10)

deg.top10.genes<-c(as.character(up.genes),as.character(down.genes))

deg.data$Label[match(deg.top10.genes,deg.data$id)]<-deg.top10.genes

ggscatter(deg.data,x="logFC",y="logP",

color="Group",

palette=c("#2f5688","#BBBBBB","#CC0000"),

size=1.5,

label=deg.data$Label,

font.label=9,

repel=T,

xlab="log2FoldChange",

ylab="-log10(p value)",)+theme_base()+

geom_hline(yintercept=1.3,linetype="dashed")+

geom_vline(xintercept=c(-1,1),linetype="dashed")

dev.off()
